# Hemolysis and Inflammatory Response to Extracorporeal Circulation during On-Pump CABG: Comparison between Roller and Centrifugal Pump Systems

**DOI:** 10.21470/1678-9741-2017-0125

**Published:** 2018

**Authors:** Andréia Cristina Passaroni, Marcello Laneza Felicio, Nelson Leonardo Kerdahi Leite de Campos, Marcos Augusto de Moraes Silva, Winston Bonida Yoshida

**Affiliations:** 1 Discipline of Cardiovascular Surgery, Department of Surgery and Orthopedics, Hospital das Clínicas da Faculdade de Medicina de Botucatu da Universidade Estadual Paulista (HCFMB-UNESP), Botucatu, SP, Brazil.

**Keywords:** Cardiopulmonary Bypass, Hemolysis, Inflammation

## Abstract

**Objective:**

To compare the perioperative incidence rates of hemolysis and inflammatory response in patients undergoing coronary artery bypass grafting with the two main types of cardiopulmonary bypass, centrifugal and roller pumps, and establish correlations among hemolytic and inflammatory changes.

**Methods:**

This was a prospective, randomized trial of 60 patients assigned to either roller pump (G1, n=30) or centrifugal pump (G2, n=30) bypass. Markers of hemolysis (serum haptoglobin, lactate dehydrogenase [LDH]) and inflammation (interleukin [IL]1ß, IL-6, and TNF-α) were measured and analyzed.

**Results:**

There was no significant between-group difference in the variables of interest. In G1, there was a positive association with IL-6 and TNF-α (*P*<0.01 and *P*<0.05, respectively). In G2, there was a positive association with LDH in the postoperative period (*P*<0.5). At 24h post-cardiopulmonary bypass, there were positive associations between LDH and IL-1ß (*P*<0.05), LDH and TNF-α (*P*<0.01), haptoglobin and TNF-α (*P*<0.05), and LDH and TNF-α (*P*<0.01) in G1, and between LDH and IL-6 (*P*<0.01), LDH and TNF-α (*P*<0.01), and LDH and IL-6 (*P*<0.01) in G2.

**Conclusion:**

There were no significant between-group differences in markers of hemolysis or inflammation. IL-6 and TNF-α were positively associated with duration of cardiopulmonary bypass in G1, while LDH was positively associated with duration of cardiopulmonary bypass in G2. The rate of significant associations between markers of hemolysis and inflammation was higher in the roller pump group (G1).

**Registration number:**

ReBEC (RBR-92b9dg).

**Table:** 

Abbreviations, acronyms & symbols	
ANOVA	= Analysis of variance
CABG	= Coronary artery bypass graft
CPB	= Cardiopulmonary bypass
CRP	= C-reactive protein
ELISA	= Enzyme-linked immunosorbent assay
FAPESP	= São Paulo State Research Foundation
ICU	= Intensive care unit
IL	= Interleukin
LDH	= Lactate dehydrogenase
MAP	= Mean arterial pressure
PO	= Postoperative
TNF-α	= Tumor necrosis factor alpha

## INTRODUCTION

For many years, investigators sought to develop devices that could replace cardiopulmonary function during cardiovascular surgery^[1]^. Cardiopulmonary bypass (CPB) allows this, but has complex effects. As the blood passes through the CPB circuit and comes into contact with synthetic materials, it undergoes mechanical insults, trauma, and cellular changes. The changes induced by these insults, including hemolytic and inflammatory alterations, can have major clinical implications^[2,3]^. To Vieira Junior et al.^[3]^, hemolysis in CPB is a result of the passage of blood through the pump rollers or the contact of blood with different surfaces at varying speeds. These authors noted that the basic requirement for developing an appropriate CPB pump would be to achieve an optimal balance between shear stress and exposure time to minimize the rate of hemolysis. In a review of core aspects of CPB, Mota et al.^[2]^ posit that bypassrelated inflammation would be caused by blood coming into contact with the operative wound (as the surgical bed is a site of interleukin [IL] -6 release), not with the non-endothelialized surfaces of the artificial CPB circuit. These authors explain that many factors during CPB, some dependent on the circuit (exposure of blood to non-physiological surfaces and conditions) and others independent (surgical trauma, ischemia-reperfusion injury, changes in body temperature, endotoxin release) are inflammatory response triggers. Within this context, the present study sought to compare the incidence of hemolysis and inflammatory response in the perioperative period of on-pump coronary artery bypass graft (CABG) surgery performed with the two most common types of CPB pumps, and to establish correlations among hemolytic and inflammatory changes.

## METHODS

We analyzed 60 consecutive patients of either sex who underwent on-pump CABG at the study hospital, between August 2013 and February 2014. The exclusion criteria were: age < 30 or > 80 years; history of other cardiovascular surgery; combined carotid surgery; preoperative anemia; patients who presented signs, symptoms and complaints suggestive of inflammation or infection in the respiratory, gastrointestinal, or genitourinary tract or other body systems (as detected by history, physical examination, presence of fever, or any abnormalities in a preoperative workup including complete blood count, C-reactive protein [CRP], chest X-ray, and urinalysis). On the day of surgery, the 60 patients were allocated via simple random assignment to groups G1 (roller pump, n = 30) or G2 (centrifugal pump, n = 30). The present study was funded by a research grant from the São Paulo State Research Foundation (FAPESP) and was approved by the Research Ethics Committee of the Faculdade de Medicina de Botucatu/UNESP (protocol CEP number 3202-2009). All patients provided written informed consent for participation. All procedures were in accordance with the ethical standards of the 1964 Helsinki declaration and its later amendments or comparable ethical standards.

Patients were monitored continuously during the operation using a Dixtal^®^ DX2010 multi-parameter monitor. Body temperature was monitored with an Ag-2000 nasopharyngeal probe (Braile Biomédica^®^ Ltda., São José do Rio Preto, SP, Brazil). In accordance with institutional CPB protocols, all patients received 1000 mg hydrocortisone after induction of anesthesia and again 4 hours after the first dose. Antibiotic prophylaxis was administered to all patients as per institutional infection control committee guidelines (1500 mg cefuroxime sodium, after induction of anesthesia, and an additional 750 mg, 4 hours after the first dose).

The roller-type circuit (adult full circuit for Console Ecobec^®^, Braile Biomédica Ltda., São José do Rio Preto, SP, Brazil) comprised a set of veno-arterial tubing, suction connectors, arterial filter, polypropylene hollow-fiber membrane oxygenator, and a conventional hemoconcentrator. Centrifugal-pump CPB used the same circuit described above, with the addition of a vortexcone centrifugal blood pump (BPX-80 Bio Pump Plus Centrifugal^®^, Medtronic) attached to the console (Console Centrífuga Bio-Medicus^®^ and Bio-Probe TX40 Flow Transducer^®^, Medtronic). After assembly of the CPB apparatus, pumps were calibrated for the CPB procedure. Upon being weaned off bypass, patients were administered dobutamine IV 3-5 µg/kg/min and noradrenaline intravenous as needed to keep mean arterial pressure (MAP) > 70 mmHg. After surgery, while still under general anesthesia, patients were transferred to the intensive care unit (ICU) for postoperative (PO) care, where they were progressively weaned off both infusions as hemodynamic status improved.

Radial artery catheterization was performed by the cutdown technique for measurement of MAP. From this catheter, blood samples were drawn after anesthetic induction but before CPB (time point M1), at 30 minutes of CPB (time point M2), and 24h PO (time point M3). Immediately after sampling, levels of two markers of hemolysis - serum haptoglobin and lactate dehydrogenase (LDH) - were measured. LDH measurement was performed in an automated Fusion^®^ analyzer, and haptoglobin measurement, in a Biospectro SP-220^®^ spectrophotometer. For analysis of inflammation, levels of I)-1β, IL-6, and tumor necrosis factor alpha (TNF-α) were measured at time points 1 and 2. For this purpose, samples were centrifuged and the plasma drawn into Eppendorf microtubes and stored at -20ºC. All kits for IL analysis were processed simultaneously, thus preventing any loss. Serum IL and TNF-α concentrations were measured by enzyme-linked immunosorbent assay (ELISA) using commercially available kits (IL-1β/1F2 Quantikine High Sensitivity^®^, TNF-α Quantikine High Sensitivity^®^, and IL-6 Duo Set^®^, R&D Systems, Minneapolis, MN, USA). All kits were processed in accordance with manufacturer instructions. CRP levels were measured in an automated Vitrus^®^ 5.1 analyzer in samples drawn at M1, M2, and M3.

For statistical analysis, the nonparametric Mann-Whitney U test was used for the variable age, and Student's t-test for body weight and all CPB variables. Goodman's test^[4]^ for contrasts within and among multinomial populations was used for the variables comorbidity and number of deaths. The EuroSCORE II stratification system was applied in all patients, with subsequent analysis of mean scores for each group. Nonparametric repeated measures analysis of variance (ANOVA) for independent samples with Dunn's multiple comparisons was used for analysis of haptoglobin and CRP. Nonparametric repeated-measures ANOVA for independent samples with Bonferroni multiple comparisons^[4]^ was used for analysis of LDH and IL levels. Spearman linear correlation coefficients were calculated to test for correlation between each of the studied markers and duration of CPB, as well as for correlations among markers of hemolysis and markers of inflammation. Using the study of Pêgo-Fernandes et al.^[5]^ as a basis, the sample size was calculated as 30 patients per group for a statistical power of 80% and a significance level of 5%.

## RESULTS

The demographic characteristics of participants were similar in the two groups (Table 1). Mean patient age was 66 years in G1 and 63 years in G2. Participants in both groups were predominantly male (73.3% in G1, 80% in G2) and had hypertension (76.7% in G1, 70% in G2). Mean patient weight was difference in presence of diabetes (*P*>0.05) or risk factors for blood transfusion (*P*>0.05).

**Table 1:** Demographic characteristics of the sample, stratified by group.

Variables		Group		*P*-value
		G1 (roller pump)	G2 (centrifugal pump)	
*Age (years)		66 (42-74)	63 (38-73)	>0.05
Weight (kg)		70.40 (11.76)	73.03 (15.07)	>0.05
Sex	Male	22 (73.3)	24 (80.0)	>0.05
	Female	8 (26.7)	6 (20.0)	>0.05
Risk Factors	Hypertension	23 (76.7)	24 (70.0)	>0.05
	No hypertension	7 (23.3)	9 (30.0)	>0.05
	Diabetic	12 (40.0)	17 (56.7)	>0.05
	No diabetic	18 (60.0)	13 (43.3)	>0.05
	Transfusion	19 (63.3)	14 (46.6)	>0.05
	No transfusion	11 (36.7)	16 (53.4)	>0.05
EuroSCORE II		0.88	0.75	>0.05

Mean morbidity and mortality risk stratification scores were 0.88 in G1 and 0.75 in G2; thus, patients in both groups were deemed to have low risk (score 0-2). There was no significant between-group difference (*P*>0.05).

Table 2 shows CPB variables for both groups. There were no significant differences between the two (*P*>0.05).

**Table 2:** Mean (standard deviation) CPB-related variables, stratified by group.

Variables	Groups		*P*-value
	G1 (roller pump)	G2 (centrifugal pump)	
Duration of CPB (min)	83.17 (31.61)	81.50 (25.50)	>0.05
Arterial flow (mL/kg/min)	3637.33 (523.21)	3767.00 (671.03)	>0.05
MAP during CPB (mmHg)	65.80 (4.67)	65.00 (5.71)	>0.05
Aortic clamping time (min)	52.77 (24.72)	47.00 (19.04)	> 0.05
Urinary output during CPB (mL)	303.67 (208.31)	312.50 (245.74)	> 0.05

CPB=cardiopulmonary bypass; MAP=mean arterial pressure; min=minutes

Table 3 shows the results of serum haptoglobin measurements in the study groups. Significant (*P*<0.05) differences between time points were observed for the variable haptoglobin, where M1 > (M2 = M3); however, there were no significant betweengroup differences in this variable (*P*>0.05).

**Table 3:** Median (range) haptoglobin levels, stratified by group and time point.

Variable	Groups	Time point			*P*-value
		M1 (pre-CPB)	M2 (post-CPB)	M3 (24h PO)	
Haptoglobin (mg/dL)	G1 (roller pump)	101.45 (20.90-231.60)^B^	9.30 (0.00-161.00)^A^	38.90 (0.00-133.80)^A^	< 0.05
	G2 (centrifugal pump)	85.50 (0.00-263.90)^B^	17.70 (0.00-248.00)^A^	32.30 (0.00-158.30)^A^	< 0.05
G1 × G2	*P*-value	*P*>0.05	*P*>0.05	*P*>0.05	

CPB=cardiopulmonary bypass; PO=postoperatively. Uppercase letters denote within-group comparisons between time points.

A denotes the lowest value measured, and Bthe highest value measured.

B the highest value measured.

**Fig. 1:**
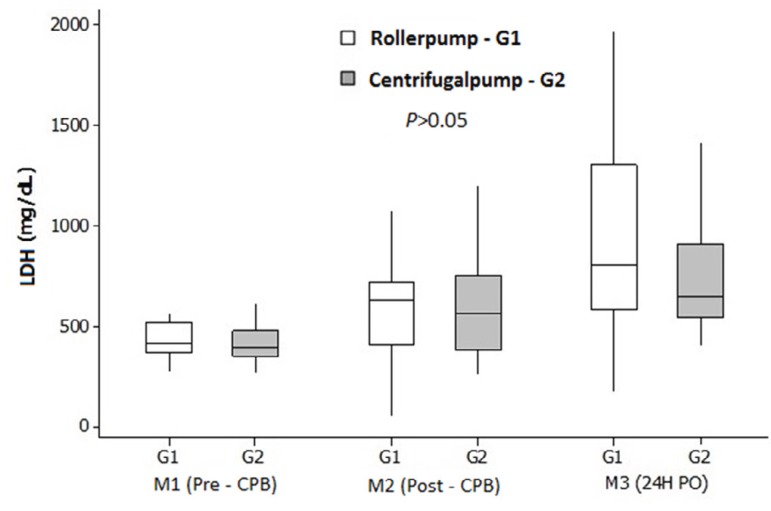
Mean (standard deviation) LDH levels, stratified by group and time point. CPB=cardiopulmonary bypass; LDH=lactate dehydrogenase; PO=postoperative

Figure 1 illustrates the results of LDH measurement. Significant (*P*<0.05) differences between time points were observed in LDH levels, where M1 = M2 < M3 in G1 and M1 < M2 < M3 in G2; however, again, there were no significant betweengroup differences in this variable (*P*>0.05).

**Fig. 2:**
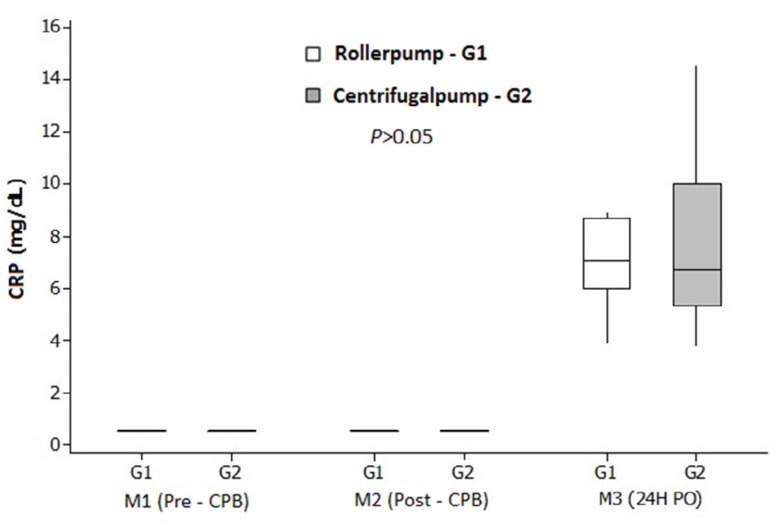
Median (range) CRP levels, stratified by group and time point. CPB=cardiopulmonarybypass; CRP=C-reactive protein; PO=postoperative

Figure 2 illustrates the results of CRP measurement. Significant (*P*<0.05) differences between time points were observed in this variable, with M1 = M2 < M3, but there were no significant between-group differences (*P*>0.05).

**Fig. 3:**
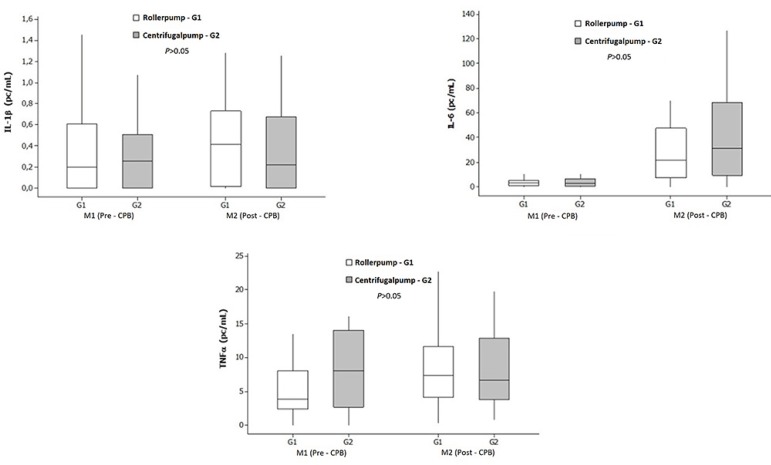
Median (range) IL-1β, IL-6, and TNF-α levels, stratified by group and time point. CPB=cardiopulmonary bypass; IL=interleukin

Figure 3 illustrates the results of analysis of markers of inflammation (IL-1β, IL-6, and TNF-α) at the pre- and post-CPB time points in both study groups. IL-1β levels did not differ significantly between groups or among time points within each group (*P*>0.05). A significant increase in IL-6 occurred post-CPB in both groups (*P*<0.01). Significant increases were also observed in TNF-α in G1 (*P*<0.01). However, there were no significant between-group differences in any of the tested inflammatory markers (*P*>0.05).

Table 4 shows a positive association between duration of CPB and LDH at 24h postoperatively in G2, as well as between duration of CPB and post-CPB IL-6 and TNF-α levels in G1.

**Table 4:** Spearman coefficients of linear association between duration of CPB and markers of hemolysis and inflammation, stratified by group.

	Groups	
Variables	G1 (roller pump)	G2 (centrifugal pump)
LDH, 24h PO	0.346	0.407*
IL-6, post-CPB	0.516**	0.304
TNF-α, post-CPB	0.382*	-0.006

* *P*<0.05;

** *P*<0.01

CPB=cardiopulmonary bypass; IL-6=interleukin-6; LDH=lactate dehydrogenase; PO=postoperatively; TNF-α=tumor necrosis factor alpha

Table 5 shows positive associations at the post-CPB time points between the variables LDH, haptoglobin and IL-1β, and IL-6 and TNF-α at the post-CPB and 24h PO time points. The frequency of associations was higher in G1 than in G2.

**Table 5:** Spearman coefficients of linear association between markers of hemolysis and inflammation, stratified by group.

Variables	Groups	
	G1 (roller pump)	G2 (centrifugal pump)
LDH post-CPB and IL-1β post-CPB	0.383*	0.318
LDH post-CPB and IL-6 post-CPB	0.315	0.793**
LDH post-CPB and TNFα post-CPB	0.555**	0.566**
Haptoglobin 24h PO and TNFα post-CPB	-0.947*	0.241
LDH 24h PO and IL-6 post-CPB	0.224	0.594**
LDH 24h PO and TNFα post-CPB	0.496**	0.272

* *P*<0.05;

** *P*<0.01

CPB=cardiopulmonary bypass; IL-6=interleukin-6; LDH=lactate dehydrogenase; PO=postoperatively; TNF-α=tumor necrosis factor alpha

The mortality rate at 48h PO was 10% in G1 (n=3; one death each due to stroke, pulmonary embolism, and cardiogenic shock) and 3.33% in G2 (n=1, due to ischemic stroke). Hence, 27 (90%) patients in G1 and 29 (96.67%) in G2 survived to discharge in the same period.

## DISCUSSION

CPB, a landmark technology that ushered in the modern age of heart surgery in the second half of the 20^th^ century, provides a means of temporarily replacing the pumping action of the heart and the ventilatory action of the lungs. It also allows recovery of blood from the operative field by suction pumps after systemic anticoagulation (intraoperative blood salvage), thus keeping the heart well-nourished while in absolute standstill induced by infusion of cardioplegic solution. The pumping action of the heart is taken over by an arterial pump (roller or centrifugal), while the ventilatory function of the lungs is replaced by an oxygenator. Biocompatible plastic tubing connects the patient, oxygenator, and arterial pump, thus allowing extracorporeal blood circulation. While some patients are fit to undergo CABG without CPB (so-called "off-pump" surgery), others require an onpump approach due to hemodynamic instability^[6]^.

Despite its significant benefits, such as providing a bloodless surgical field, CPB is associated with postperfusion syndrome, a condition whereby biochemical mediators trigger coagulopathy (through activation of the complement system), left ventricular dysfunction and myocardial ischemia (mediated by IL-6 and IL8), fibrinolysis (caused by free radicals), vasoplegia (associated with kinins), and cell death (through TNF-α action), as well as generation of reactive oxygen species as a result of ischemia and reperfusion. This combination of factors may lead to increased susceptibility to infection, potentially critical multiple-organ dysfunction, and even death^[7]^.

CPB can also cause hemolysis, either because of poor calibration of the arterial pump or occasionally because of the prolonged duration of the procedure. The most specific acutephase marker of hemolysis is serum haptoglobin. Circulating plasma haptoglobin is responsible for binding and transporting free hemoglobin from the circulation to the liver, spleen, and bone marrow, where hemoglobin is reprocessed after erythrocyte lysis. Erythrocytes also contain the enzyme LDH. LDH levels increase at the onset of cell destruction, including erythrocyte lysis (*e.g*., due to mechanical heart valve dysfunction, acute myocardial infarction, chest trauma, or CPB)^[8]^. Thus, LDH can be used as a marker of hemolysis, although it is nonspecific for this purpose. After rupture of the erythrocyte membrane, the erythrocyte stroma remains in the circulation, where it acts as a proinflammatory agent; thus, hemolysis accentuates the inflammation that occurs in CPB.

When hemolysis is particularly intense, haptoglobin levels decline markedly, and may even become undetectable. This was observed in the present study: haptoglobin concentrations declined after CPB, probably due to formation of haptoglobinhemoglobin complexes, which prevent renal loss of hemoglobin^[9]^. However, there was no significant betweengroup difference. Recovery of haptoglobin levels was observed at the 24h PO time point. In a previous comparison of roller *vs.* centrifugal pumps in a sample of 27 patients, Pêgo-Fernandes et al.^[5]^ failed to find any significant between-group difference in haptoglobin levels when the duration of CPB was < 110 minutes. In an *in vitro* experiment, Bennett et al.^[10]^ found that roller pumps were associated with marked hemolysis as compared to centrifugal pumps. Upon erythrocyte lysis, LDH is released into the circulation, and plasma LDH levels rise substantially. In the present study, elevated LDH levels were found after CPB (specifically, up to 24h postoperatively), but there was no significant between-group difference. Patients with a longer duration of CPB exhibited higher LDH levels when allocated to the centrifugal pump group, and patients in both groups exhibited a positive association of LDH levels with inflammatory mediators at the post-CPB and 24h PO time points. In a comparative study, Keyser et al.^[11]^ found no difference between pump types, although levels of this marker were elevated after CPB and remained elevated up to 12h PO. Yoshikai et al.^[12]^ also analyzed LDH in 29 patients in a comparative study of CPB pumps, but failed to find any significant difference between pump types. In a meta-analysis, Saczkowski et al.^[13]^ included randomized clinical trials comparing CPB pump types and found no differences in the variables of interest. The authors expected to find evidence of insults with all pump types; however, this was not the case. In the present study, we found that hemolytic responses occurred with both types of CPB pump at the time points of assessment, but with no significant between-group differences.

ILs mediate and regulate inflammatory and immune reactions. Although there are several proinflammatory cytokines, the present study focused on IL-1β, IL-6 and TNF-α. Even after brief periods of CPB, the inflammatory response to extracorporeal circulation immediately triggers a variety of immune reactions. In the present study, with a relatively short duration of CPB, inflammation occurred in both groups at the time points of assessment, but there was no significant between-group difference.

IL-1β levels usually rise after CPB. However, they are often undetectable due to hemodilution^[14]^. In the present study, there was no significant difference in levels of this IL across time points or groups. IL-6 levels are known to rise 2 to 4 hours after surgical incision. In patients who have undergone CPB, levels of this marker are elevated even after hemodilution^[14]^. In the present study, this phenomenon was observed in both groups at the time points of assessment, but with no significant between-group difference. Patients in G1 with a longer duration of CPB exhibited higher IL-6 levels. In a study of 41 patients, Ashraf et al.^[15]^ found that centrifugal pumps induced more severe inflammation than roller pumps, as demonstrated by significantly higher IL-6 and C5b levels in the centrifugal group, demonstrating the proinflammatory nature of CPB. TNF-α accounts for many of the systemic complications and severe infections seen after this procedure. In the present study, TNF-α levels were elevated at the post-CPB time point in G1, but there was no significant difference between groups. Among patients with a longer duration of CPB, levels of this cytokine were significantly higher in G1. Baufreton et al.^[16]^, in a study of 29 patients, also showed that centrifugal pumps induced worse inflammation than roller pumps; however, IL-6 and TNF-α levels were similar in both groups, with no significant difference. Mlejnsky et al.^[17]^ reported that the use of centrifugal pumps during CPB with prolonged hypothermic arrest is associated with a reduced inflammatory response as compared to roller-pump CPB.

It is important to note that constitutive production of cytokines occurs in the human body, whereby specialized cells express a baseline level of these proteins under normal circumstances (in the present study, the pre-CPB time point can be considered as this baseline for the cytokines of interest). It is understood that any agent or factor can trigger an inflammatory process, including cell necrosis. The liver responds to the inflammatory process by synthesizing acute-phase protein, CRP, which is a known marker of coronary heart disease risk in ill patients^[18]^. This protein is related to infectious complications, as well as to the inflammatory response induced by CPB; in this setting, its levels may increase up to 6 hours postoperatively, and correlate with the severity of inflammation. In the present study, the increase in CRP at the 24h postoperative time point suggested post-CPB inflammation, but without a significant difference between groups. Cremer et al.^[19]^ report that increased IL-6 levels promote circulatory and metabolic instability, which leads to CRP release, stimulating further tissue inflammation.

In the present study, we also sought to address whether correlations exist between markers of hemolysis and inflammatory mediators at the post-CPB and 24h PO time points. The immune system responds to trauma with varying degrees of severity by releasing molecular compounds, including those of the complement system, which stimulate release of proinflammatory cytokines such as IL-1β, IL-6 and TNF-α. Hemolysis may occur through three distinct mechanisms: natural selection in the spleen; physical or chemical imbalances (generally pathological); or exposure of cells to mechanical trauma^[3,20]^. According to Kameneva et al.^[21]^, the intensity of hemolysis depends on the flow rate of suction, on the degree of blood viscosity, on the duration of extracorporeal perfusion, and on the storage time of the packed red blood cells used in perfusate or otherwise during the CPB procedure. The mechanical hemolysis associated with CPB pumps is of the intravascular type, which causes cell lysis (particularly of erythrocytes) with subsequent release of hemoglobin into the blood plasma. With roller pumps, cell trauma may occur when the rollers are poorly calibrated or set. In centrifugal pumps, hemolysis may occur when there is excessive negative pressure driving blood flow^[22]^. Pohlmann et al.^[23]^ reported that suction during CPB is a major factor in CPBassociated hemolysis, and that the negative pressure exerted by the pump and exposure of blood to air combine to make this hemolysis more severe.

As erythrocytes rupture, their contents and remnants (the erythrocyte stroma) remain in the bloodstream, inducing an acute inflammatory response with activation of inflammatory mediators such as the complement system, which, in turn, stimulates IL-1β, IL-6 and TNF-α secretion. In our sample, we observed a positive association between haptoglobin and LDH with IL-1β, IL-6, and TNF-α at the post-CPB and 24h PO time points, denoting induction of inflammation in both groups. The frequency of this association was higher in the G1 group, both at post-CPB and at 24h PO. The roller-type pump used in this study contained four main rollers (one arterial and three for suction); compounded by variations in the blood volume present in the venous reservoir, this may account for the greater frequency of the aforementioned correlations in G1^[23,24]^. As the centrifugal CPB pump has no arterial roller and contains only three rollers (all for suction) and blood is impelled by centrifugal force, maintaining a stable blood volume in the venous reservoir, the frequency of these correlations was lower in G2. Cell trauma was observed with both CPB pumps, but more severely with the roller pump (G1), as demonstrated by the frequency of associations.

According to Mota et al.^[2]^, the onset of hemolysis and inflammation also depends on the duration of CPB, the conduction of CPB and the material used in the process. According to Dienstmann and Caregnato^[25]^, stress that conducting perfusion with good cardiac output, brief duration of CPB whenever possible, strict control of initial venous drainage and hemodilution, and proper oxygenation and acid-base balance could minimize hemolysis and inflammation. These authors also noted the relevant correlation between inflammatory response and hemolysis in the postoperative period of patients who undergo on-pump cardiovascular surgery, as the possibility of multisystem complications could have repercussions for duration of mechanical ventilation and length of ICU stay. In the present study, the release of inflammatory mediators during CPB was clearly unrelated to age, sex, preoperative cardiac function, or even type of cardiovascular surgery. Both groups were homogeneous in terms of demographic characteristics, operative technique and perfusion technique, thus enabling comparison of the outcomes of interest. All patients underwent morbidity and mortality risk stratification via the EuroSCORE II system, which showed that patients in both groups were at low risk of adverse outcomes (scores in the range of 0-2). Both pump types tested proved efficient and safe during CPB, as demonstrated by the analyzed parameters.

In this study, as in most cardiovascular surgery procedures, hydrocortisone was administered in an attempt to minimize the inflammatory effects of CPB. Although patients received this corticosteroid both before CPB (M1) and during CPB (M2), levels of inflammatory markers were elevated in both study groups.

In a meta-analysis, Ali-Hassan-Sayegh et al.^[26]^ showed that prophylactic corticosteroids could reduce the complication rate significantly and improve clinical outcomes in patients undergoing CABG, and could be considered both safe and effective. Zakkar et al.^[27]^ states that hemolysis, ischemiareperfusion injury, and neutrophil activation during CPB play critical roles in oxidative stress and proinflammatory activation, which, in turn, may lead to multiple-organ dysfunction. These investigators believe the administration of antioxidant agents during surgery could mitigate the negative effects of CPB.

Although we deemed our sample size according to Pêgo-Fernandes et al.^[5]^, we believe it was still small enough to constitute a limitation, and that further research with larger samples is warranted. Due to the small sample size per group and the relatively short, similar duration of CPB in both groups, the study may have been underpowered to detect differences between pump types. Although the routine administration of vasopressors follows standardized protocols at our facility, the lack of data on vasopressor dosages used in each group constitutes a limitation of the present study. Future studies by our group in this line of research will include analysis of vasopressor dosage and larger sample sizes.

## CONCLUSION

In conclusion, both CPB pump types tested in this study induced hemolysis and inflammation concurrently. However, there were no significant differences in markers of hemolysis or in inflammatory mediators between the centrifugal pump and roller pump groups. Positive associations were found between LDH and duration of CPB at 24h postoperatively in G2 (centrifugal pump) and between IL-6 and TNF-α and duration of CPB immediately after CPB in G1 (roller pump). Positive associations were also found among markers of hemolysis and markers of inflammation in G1 and G2, both post-CPB and at 24h postoperatively; however, the frequency of such associations was higher in G1.

**Table:** 

Authors' roles & responsibilities	
ACP	Conception and design; analysis and interpretation; data collection; writing the article; critical revision of the article; statistical analysis; overall responsibility; final approval of the article
MLF	Data collection; writing the article; overall responsibility; final approval of the article
NLKLC	Data collection; writing the article; overall responsibility; final approval of the article
MAMS	Conception and design; analysis and interpretation; writing the article; critical revision of the article; statistical analysis; overall responsibility; final approval of the article
WBY	Analysis and interpretation; writing the article; critical revision of the article; statistical analysis; overall responsibility; final approval of the article
